# Expanding the Role of Mechanical Circulatory Support in Transcatheter Aortic Valve Replacement for Cardiogenic Shock

**DOI:** 10.1016/j.jscai.2025.104010

**Published:** 2025-10-14

**Authors:** Dan Haberman, Uri Landes, Jacob George

**Affiliations:** a Heart Center, Kaplan Medical Center, Rehovot, Israel; b Hebrew University, Jerusalem, Israel; c Section of Interventional Cardiology, MedStar Washington Hospital Center, Washington, District of Columbia; d Department of Cardiology, Bnai-Zion Medical Center, Haifa, Israel; e Technion University, Haifa, Israel

**Keywords:** cardiogenic shock, mechanical circulatory support, transcatheter aortic valve replacement

Severe aortic stenosis (AS) complicated by cardiogenic shock (CS) represents one of the most formidable challenges in structural cardiology. The natural history of this condition is dismal: medical therapy alone carries exceedingly high mortality, and surgical valve replacement is seldom feasible in acutely unstable patients. Transcatheter aortic valve replacement (TAVR) has transformed the landscape for treating AS, offering a minimally invasive solution for patients deemed inoperable. However, the hemodynamic instability of patients in shock often precludes safe periprocedural management. In this precarious clinical scenario, mechanical circulatory support (MCS) is explored as a potential bridge, stabilizing hemodynamics, preserving organ perfusion, and extending the window of opportunity for treatment.

Left ventricular (LV) dysfunction lies at the core of AS progression to symptomatic heart failure (HF) and CS. The narrowed aortic valve imposes a chronic pressure overload, leading initially to concentric hypertrophy in an attempt to maintain systolic function. Over time, this adaptation gives way to impaired LV compliance, diastolic dysfunction, and eventual systolic failure. When the ventricle can no longer overcome the elevated afterload, afterload mismatch, stroke volume, and cardiac output collapse. In patients with reduced LV ejection fraction, the weakened myocardium cannot sustain forward flow, culminating in systemic hypoperfusion, pulmonary congestion, and the clinical picture of HF or CS. Once this downward spiral begins, immediate hemodynamic support is often required.

In this issue of *JSCAI*, Khan et al^1^ provide timely, national-level insights into the use of MCS as a bridge to TAVR in this high-risk population. Drawing on more than 250,000 TAVR cases from the Vizient Clinical Database (2016-2023), the authors identified 575 patients who received MCS prior to TAVR. Their analysis yields several important observations:

1. Rising MCS utilization: Use of MCS-TAVR has increased nearly 4-fold over the study period, from 3.7% of TAVR cases in shock in 2016 to nearly 15% by 2023. This trend reflects both expanding recognition of shock, advances in technologies, and increased operator familiarity with percutaneous support devices.
2. Outcomes: In-hospital mortality reached 21%, higher than prior reports of unselected TAVR patients with CS (∼10%), but remarkable, given the acuity of illness. Nearly 80% of these critically ill patients survived hospitalization, underscoring the potential benefit of hemodynamic stabilization before valve intervention.
3. Complications: High rates of acute kidney injury (78%) and major bleeding (53%) reflect both patient frailty and morbidity and the invasive nature of MCS. Functional recovery remained limited, with only 17% discharged home.

These sobering yet encouraging data confirm that MCS-facilitated TAVR is feasible, yet far from being benign. Importantly, they suggest that MCS should not be considered a last-ditch “rescue” for periprocedural collapse but rather a proactive strategy in carefully selected patients.

A range of MCS devices is available, each with distinct advantages and limitations: Intra-aortic balloon pump is widely available, less costly, and associated with low complication rates but provides only modest hemodynamic augmentation; Impella (Abiomed) offers direct LV unloading and increased output, with the caveats of a higher rate of vascular complication and challenges in inserting in patients with severe AS. Venoarterial extracorporeal membrane oxygenation delivers robust circulatory and respiratory support in profound shock or multiorgan failure but carries significant risks, including bleeding, vascular injury, and increased LV afterload. Device selection should be individualized according to shock severity, ventricular function, procedural factors, and institutional expertise. Moreover, it would be critical to balance their potential disastrous complications against the grim prognosis in patients with CS and AS, which could only be tested in a randomized trial.

Perhaps the most critical unanswered question is when to deploy MCS. Early initiation may protect against end-organ injury and create a more stable platform for TAVR. On the other hand, earlier support exposes patients to risks of bleeding, vascular complications, and device-related issues. Observational studies suggest that the need for MCS itself is an independent predictor of mortality after TAVR, likely reflecting the severity of the underlying clinical scenario. Without clear evidence-based data, decisions must be individualized—balancing hemodynamic instability, comorbid burden, and realistic goals of care. A multidisciplinary heart team approach is essential.

Finally, yet importantly, better upstream surveillance after AS patients and on time treatment acquisition are expected to be the most important measures of treating these patients. Although a propensity analysis comparing patients not supported by MCS and those not requiring it could have further strengthened the validity of the findings, especially given the size of the database, the present work nevertheless provides valuable insights.

To move beyond descriptive data, the field requires prospective registries and, ideally, randomized studies. Comparative analyses of device strategies, timing, and weaning protocols are urgently needed. Incorporating physiologic variables such as SCAI SHOCK stage,^2^ invasive hemodynamics, and biomarkers could refine patient selection and prognostication. Importantly, collaboration across interventional cardiology, advanced HF, and critical care disciplines is indispensable.
